# Clinical and Genetic Analysis of a Family With Sitosterolemia Caused by a Novel ATP-Binding Cassette Subfamily G Member 5 Compound Heterozygous Mutation

**DOI:** 10.3389/fcvm.2022.887618

**Published:** 2022-04-26

**Authors:** Ming-fang Shen, Ya-nan Hu, Wei-xiang Chen, Li-sheng Liao, Min Wu, Qiu-yan Wu, Jian-hui Zhang, Yan-ping Zhang, Jie-wei Luo, Xin-fu Lin

**Affiliations:** ^1^Fujian Provincial Hospital, Shengli Clinical Medical College of Fujian Medical University, Fuzhou, China; ^2^Pediatrics Department, Fujian Provincial Hospital, Fuzhou, China; ^3^Department of Hematology, Fujian Provincial Hospital, Fuzhou, China; ^4^Department of Traditional Chinese Medicine, Fujian Provincial Hospital, Fuzhou, China

**Keywords:** sitosterolemia, sterolin-1 protein, *ABCG5*, mutation, bioinformatics analysis

## Abstract

Sitosterolemia (OMIM ##210250), also known as phytosterolemia, is a rare autosomal recessive disorder caused by mutations in the ATP-binding cassette subfamily G member 5 (*ABCG5*) or member 8 (*ABCG8*) genes. This leads to abnormal functions of the transporter sterolin-1 protein encoded by *ABC*G5 and sterolin-2 protein encoded by *ABC*G8, respectively, which can hinder the formation of stable *ABCG5*/*G8* heterodimers, decreasing its ability to transport sterols. As a result, phytosterols in tissue or plasma are significantly increased, leading to early onset atherosclerosis-related diseases and xanthelasma of tendons and skin. In this study, whole exome sequencing was performed on a Chinese Han proband with sitosterolemia to capture the target gene and screen for suspected pathogenic mutations. Sanger sequencing of the family members was performed to verify the relationship between family genetics and phenotypes. The structural and functional changes in the transporter sterolin-1 protein after the responsible mutation were predicted using bioinformatics analysis. A novel compound heterozygous mutation in the *ABCG5* gene (NM_022436) was identified in a proband with sitosterolemia, one of which was inherited from the father: c.296T >G (p.M99R), and one from the mother: c.−76 C >T. SIFT, Polyphen2, and Mutation Taster software predicted that p.M99R may be the responsible variant and a novel variant. RNAFold software predicts that c.−76 C >T may affect the transcriptional information or the binding of RNA binding proteins by regulating the structure of RNA, and ultimately affect gene transcription or RNA stability and translation. Swiss model software predicts that the amino acid sequence around p.M99R is highly conserved, and p.M99R leads to instability of the tertiary structure of the *ABCG5*/*ABCG8* heterodimer. GPS 5.0 predicted that M99R affects the phosphorylation of nearby amino acid sequences, and DUET and VarSite software predicted that M99R affects the stability of sterolin-1 and cause disease. The p.M99R and c.−76 C >T mutations led to the formation of unstable heterodimers, which disturbed sterol absorption and excretion *in vivo*. The compound heterozygous variants c.296 T >G (p.m99r) and C.−76 C >T on exon 3 of *ABCG5* in this family may be the molecular genetic basis of sitosterolemia.

## Introduction

Sterols are important natural active substances that are widely present in many living things, but the type of sterol used can be different- thus fungi use ergosterol, plants use phytosterols and mammals use cholesterol (1). Cholesterol is a sterol, can be synthesized internally, but phytosterols are not synthesized in the human body and can only be obtained through the diet. Phytosterols are components of plant cell membranes, existing mainly in vegetable oil, grains, fruits, vegetables, and nuts, with cereal sterol being the most abundant (2). Under normal conditions, the absorption of cholesterol by the human body is approximately 50%, while only a negligible proportion of phytosterols (especially xenosterols) are absorbed (<5%); they are almost discharged into the intestinal cavity and bile, and are kept at a very low levels by the action of ABCG5/ABCG8.

Sitosterolemia (OMIM ##210250), also known as phytosterolemia, is a rare autosomal recessive disorder. The clinical manifestations are similar to those of familial hypercholesterolemia, immune thrombocytopenia, and Evans syndrome. Phytosterols in the blood of normal individuals account for only 0.2%. As the plasma sitosterol content cannot be detected by routine examination, sitosterolemia can be easily misdiagnosed or missed (3, 4). Sitosterolemia is caused by mutations in ATP-binding cassette subfamily G member 5b (*ABCG5*) or *ABCG8* (5). According to the Exome Aggregation Consortium (ExAC), a public gene bank, there may be 1 in every 220 people in the general population. Individuals develop gene loss-of-function (LOF) mutations in *ABCG5* or *ABCG8* (6).

## Materials and Methods

### Subjects

The proband was a 7-year-old male of Han nationality from Fujian, China. The main clinical manifestations were several raised nodules of different sizes on the elbow joints, finger joints, and ankle joints, with the largest being approximately 1 × 1.5 cm, pale yellow with borders clear, firm, and non-tender. A routine biochemical full set was re-examined in external hospitals several times, which indicated that blood lipid indicators, such as cholesterol and low-density lipoprotein, were significantly increased. The parents were not consanguineous, and there was no family history of coronary heart disease or increased blood lipid levels. The blood phytosterols spectrum and gene sequencing of the family members were investigated based on clinical manifestations, examination results, and family history of the child. This study was approved by the Ethics Committee of Fujian Provincial Hospital, and all family members or guardians who participated in this study signed an informed consent form.

### Determination of Plasma Phytosterols by Gas Chromatography—Mass Spectrometry

Next, 0.1 ml of plasma was placed in a 10 ml centrifuge tube, 0.1 ml of mixed standard solution (including the following standards: squalene, cholestanol, desmosterol, lathosterol, campesterol, stigmasterol, stiosterol) was added, then 1 ml 1 mol/L Potassium hydroxide ethanol solution were also added, vortexed and mixed, sealed with a stopper, and saponified at 60°C for 60 min. After saponification, the solution was cooled to room temperature, 1 ml of deionized water and 3 ml of n-hexane were added, vortexed for 2 min, centrifuged at 3000 r/min for 10 min, and the supernatant (n-hexane layer) was transferred to a 10 mL centrifuge tube. 3 ml of n-hexane was added to the lower aqueous phase, vortexed and mixed, and centrifuged at 3000 r/m for 10 min. The supernatant was taken out and combined, and air dried with high-purity nitrogen at 40°C. 0.1 ml of derivatization reagent (BSTFA-TMCS = 99:1) was added to the residue and placed in a 60°C oven for 60 min. After the derivatization reaction, 1 μl of the sample was directly taken for analysis by gas chromatography—mass spectrometry (GC-MS) (GCMS-QP2010Plus, Shimadzu Corporation, Japan; Beijing Fu-you-Long-hui Genetic Disease Clinic assists in completing GC-MS).

### Mapping and Screening of Mutant Genes in Proband and Family Members

Next-generation sequencing of the proband was performed, and DNA capture chip with multiple genes was used to enrich multiple gene fragments, comprising the target genes *ABCG5*, *XYLT2*, *NBAS*, *SLC6A19*, *ERCC6*, *ATP7B*, and *BMP1*. Protein function prediction was performed using SIFT, Polyphen2, and MutationTaster software. Sanger sequencing was used for verification, and primers for target sequences were designed using Primer Premier 5.0. The amplified fragment length of the target sequence where the suspected mutation point *ABCG5*(NM_022436) c.296T >G (p.M99R) was 492 bp. The following primers were used: forward, GAAGGAATGGGCAAGCGTAGG; reverse, TCATGCCTGCACAGAGGGGTCT. The annealing temperature was set to 58°C. The amplified fragment of the target sequence where another suspicious mutation, c.-76 C >T, was located was 369 bp. The following primers were used: forward, ACAAACGTGTGTGTTCTGCC; reverse, TGCCTTACCTGACGCTGTAG. The annealing temperature was set to 57°C. The primers were synthesized by Wuhan Kangshengda Medical Laboratory and the PCR products were sequenced according to the standard procedure of BigDye Terminator v3.1 kit.

### Biological Information Analysis Method

Swiss model and Chimera were used to predict the effect of M99R mutation on protein tertiary structure;Sift and Polyphen software were used to analyze the conservation of amino acid sequence near M99R; GPS 5.0 was used to predict the effect of M99R mutation on phosphorylation modification of nearby amino acid sequence. DUET software was used to predict the effect of M99R mutant on the stability of ABCG5 protein. The pathogenicity of M99R mutant was predicted by VarSite software. RNAFold software was used to predict the binding of transcription factors at ABCG5 c.-76C site and analyze the conservatism.

## Results

### Evaluation of the Clinical Data of the Probands and Family Members

**FIGURE 1:**
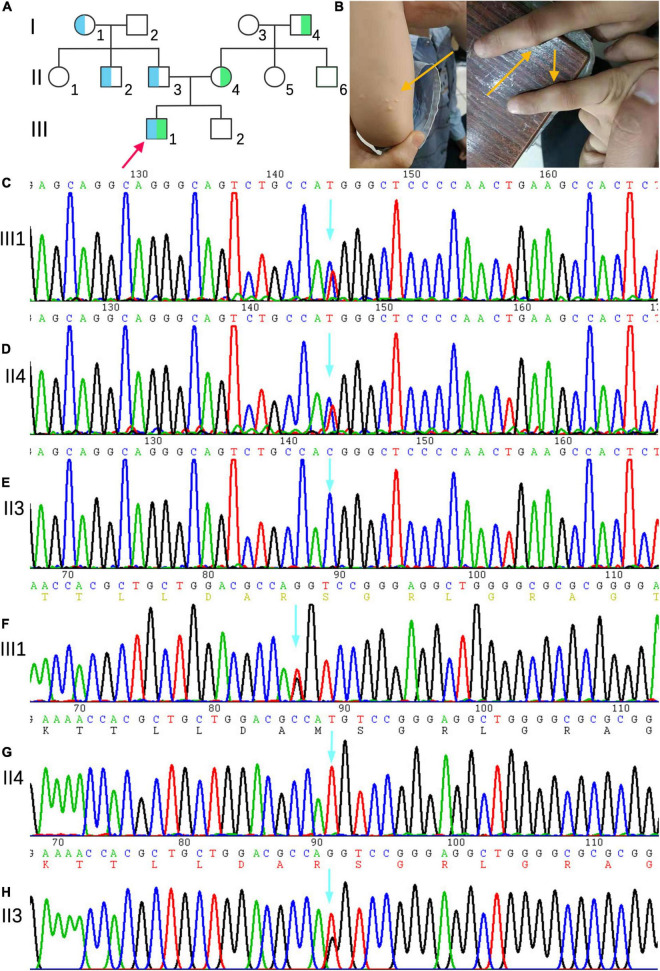
**(A)** Family genetic pedigrees map for sitosterolemia. Blue indicates a carrier of the mutation of *ABCG5*(NM_022436) c.296 T >G (p.M99R) and green indicates a carrier of the mutation of *ABCG5* c.-76 C >T. the arrow indicates the proband, the square indicates males, and circle indicates female. **(B)** The proband’s joint skin xanthoma. **(D–E)** Sanger sequencing showed that proband III1 carried the c.-76 C >T heterozygous variant inherited from mother (II4). **(F–H)** Sanger sequencing map showing that proband III1 carried the c.296T >G (p.M99R) heterozygous variant inherited from father (II3).

The family consisted of 12 members, comprising seven males and five females, wherein only one proband had sitosterolemia (Figure 1A). A 7-year-old proband (III1) was found to have several xanthomas of various sizes in the elbow, knuckle, and ankle joints (Figure 1B). Physical examination showed that several protuberant nodules of different sizes could be found in the elbow, finger and ankle of the child, with a maximum of about 1 × 1.5 cm, light yellow, clear boundary, hard, and no tenderness. After admission, the results of routine blood examination including platelet count and volume, liver and kidney electrolytes, and blood glucose tests were all normal. Tests for autoantibodies and anti-O antibodies were negative. The immune panel, blood cell sedimentation rate, and C-reactive protein level were also normal. Thyroid function tests showed no abnormalities in TSH, T3, and T4 levels. The electrocardiogram (ECG) findings were unremarkable. Full set of blood lipids: Serum total cholesterol (TC) 15.22 mmol/l (normal is <5.18 mmol/l), triglyceride (TG) 1.68 mmol/l (normal is <1.7 mmol/l), low density lipoprotein cholesterol (LDL-C) 13.28 mmol/l (normal is 1.56–3.37 mmol/L), high-density lipoprotein cholesterol (HDL-C) 1.18 mmol/l (normal is 1.29–1.55 mmol/l), apoprotein A (apoA) 1.02 g/l (normal is 1.2–1.6 g/l), apoprotein B (apoB) 3.57 g/l (normal is 0.6–1.1 g/l). Phytosterols spectrum report: squalene 0.41 μmol/L (normal 0.30–4.00 μmol/L), Cholesterol 23.48 μmol/L (normal 0.01–10.00 μmol/L), Desmosterol 2.83 μmol/L (normal 0.30–5.00 μmol/L), Lathosterol 0.64 μmol/L (normal 0.01–12.50 μmol/L), Campesterol 34.79 μmol/L (normal 0.01–10.00 μmol/L), Stigmasterol 5.74 μmol/L (normal 0.10–8.50 μmol/L), Sitosterol 280.29 μmol/L (normal 1.00–15.00 μmol/L) (Table 1). Therefore, the proband was diagnosed with sitosterolemia in combination with clinical manifestations, laboratory tests, exclusion of familial hypercholesterolemia and cerebral tendon xanthoma, and subsequent genetic sequencing.

**TABLE 1:** Biochemical indicators of family members with sitosterolemia.

Item		III1	II3	II4	II2	Reference value
Blood lipid	Triglyceride, mmol/L	1.18	1.16	1.14	1.11	0–1.7
	Total cholesterol, mmol/L	15.22↑	4.02	3.43	3.23	0–5.17
	High-density lipoprotein, 5mmol/L	1.18	1.16	1.41	1.21	1.16–1.5
	Low-density lipoprotein, mmol/L	13.28↑	2.55	1,76	1.55	1.56–3.37
	Apolipoprotein A1, g/L	1.02	1.47	1.59	1.38	1.2–1.6
	Apolipoprotein B, g/L	3.57 ↑	0.68	0.82	0.98	0.8–1.05
	APO-A1/APO-B	3.5	0.46	0.51	0.71	0.43–0.83
phytosterol spectrum	Squalene, μmol/L	0.41	2.12	1.65	0.65	0.30–4.0
	Cholesterol, μmol/L	23.48↑	7.21	4.32	1.32	0.01–10.0
	Desmosterol, μmol/L	2.83	3.23	4.33	2.21	0.30–5.00
	Lathosterol, μmol/L	0.64	5.67	7.54	1.23	0.01–12.50
	Campesterol, μmol/L	34.79↑	5.67	6.54	2.43	0.01–10.00
	Stigmasterol, μmol/L	5.74	4.32	6.43	5.43	0.10–8.50
	Sitosterol, μmol/L	280.2↑	10.23	11.2	3.4	1.00–15.00
Liver function of blood biochemistry	Total protein, g/L	75.6	78.4	67.2	72.3	60–80
	Albumin, g/L	41.5	50.4	39.5	44.3	35–55
	Globulin, g/L	34.1	28	26	22	20–30
	A/G	1.22	1.8	1.5	2.0	1.2 –2.4
	Total bilirubin, μmol/L	5.7	11.08	10.2	7.9	5.1–19
	Direct bilirubin, μmol/L	1.9	8.1	6.8	2.2	1.7–6.8
	Indirect bilirubin, μmol/L	3.8	3.01	3.3	3.8	0–12
	Alanine aminotransferase, U/L	16.1	43.9	30.3	23.2	5–40
	Aspartate transferase, U/L	27.4	25.5	29.3	23.4	8–40
Renal function of blood biochemistry	Blood urea nitrogen, mmol/L	2.39	4.22	3.34	2.58	2.86–8.2
	Creatinine, μmol/L	42.7	73.3	63.4	60.2	62–116
	Uric acid, μmol/L	281.4	234.3	278.4	244.5	208–428
Heart function of blood biochemistry	Creatine kinase, U/L	128.6	78.1	99.5	60.1	50–310
	Creatine kinase isoenzyme, U/L	27.3	22.1	15.6	21.1	0–25
	Erythrocyte sedimentation rate, mm/h	11	9	13	7	0–15
Routine blood examination	White blood cell × 10^9^/L	6.05	4.21	7.23	2.34	4–10
	Hemoglobin,g/L	130	126	133	130	120–140
	Platelet count × 10^9^/L	264	241	272	243	125–350
	Platelet volume,fl	10.4	9.8	10.0	7.9	7–11

*APO-A1/B, Apolipoprotein A1/B; A/G, Ratio of albumin to globulin.*

The child was treated with ezetimibe 5mg QD for 2 months after diagnosis, and had a low phytosterols diet at the same time. Re-examination of phytosterol spectrum showed: Sitosterol spectrum report: squalene 0.48 μmol/L (normal 0.30–4.00 μmol/L), Cholesterol 12.77 μmol/L (normal 0.01–10.00 μmol/L), Desmosterol 2.90 μmol/L (normal 0.30–5.00 μmol/L), Lathosterol 0.70 μmol/L (normal 0.01–12.50 μmol/L), Campesterol 95.17 μmol/L (normal 0.01–10.00 μmol/L), Stigmasterol 6.58 μmol/L (normal 0.10–8.50 μmol/L), Sitosterol 93.18 μmol/L (normal 1.00–15.00 μmol/L). The cholestanol and stiosterol were significantly lower than before, but campesterol was increased. Blood lipid reexamination: Serum TC 4.86 mmol/l (normal is <5.18 mmol/l), TG 0.83 mmol/l (normal is <1.7 mmol/l), LDL-C 2.24 mmol/l (normal is 1.56–3.37 mmol/l) L), HDL-C 1.78 mmol/l (normal is 1.29–1.55 mmol/l), apoprotein A 1.5 g/l (normal is 1.2–1.6 g/l), apoprotein B 0.87 g/l (normal is 0.6–1.1 g/l). The previously elevated TC and LDL-C decreased to normal. The treatment of ezetimibe and low phytosterols diet has a significant effect on the improvement of the child’s condition.

Except for the slightly higher serum TC and TG levels of I2, I4, and II5, the lipid metabolism-related indicators of other family members, sitosterol test spectrum, routine blood examination including platelet count and volume, liver and kidney electrolytes, cardiac function, and blood glucose tests were normal. Tests for autoantibodies and anti-O antibodies were negative. Blood cell sedimentation rate and C-reactive protein levels were normal. Thyroid function and ECG findings were normal.

### Gene Mutation Analysis of the Probands and Family Members

Next-generation sequencing analysis showed that two heterozygous mutations were found in the *ABCG5* gene (NM_022436) in the proband, forming a compound heterozygous mutation. The two mutation points were from the parents, one of which was inherited from the father: c.296T >G (p.M99R), and the other from the mother, and the reference nucleotide sequence hg19, in which thymine (T) at c.296 of exon 3 is replaced by guanine (G), resulting in the replacement of amino acid methionine at position 99 of the coding region by arginine. This mutation was not included in the Human Gene Mutation Database (HGMD), Clinvar Database, MAF population frequency (MAF is the maximum value of ExACALL, esp6500, 1000G frequency), or gnomAD population in East Asia. The predicted scores of SIFT, polyphen2 and mutation taster were 0.006, 0.777, and 0.956, respectively. Referring to the interpretation guide of ACMG gene mutation, this locus meets four requirements (PM1, PM2, PP3, and PP4) and can be graded as a possible pathogenic mutation. The c.-76 C >T mutation in the mother was located in the vicinity of exon 3, and the cytosine (C) of c.-76 was replaced by thymine (T). This variant was not included in the HGMD, Clinvar, and East Asian gnomAD populations. The population frequency of MAFs for this locus was 0.0002. Referring to the interpretation guide of ACMG gene mutations, this locus meets two requirements (PM2, PP4), whose grading evaluation was of unknown significance. The proband’s mother (II4) and grandfather (I4) carried C.-76 >T heterozygotes, whereas the proband’s grandmother (I1), uncle (II2), and father (II3) carried c.296 T >G (p.M99R) heterozygotes (Figures 1C–H). III2, I2, II1, II5, and II6 were wild-type, without carrying the above two heterozygotes.

### Biological Information Analysis

**FIGURE 2:**
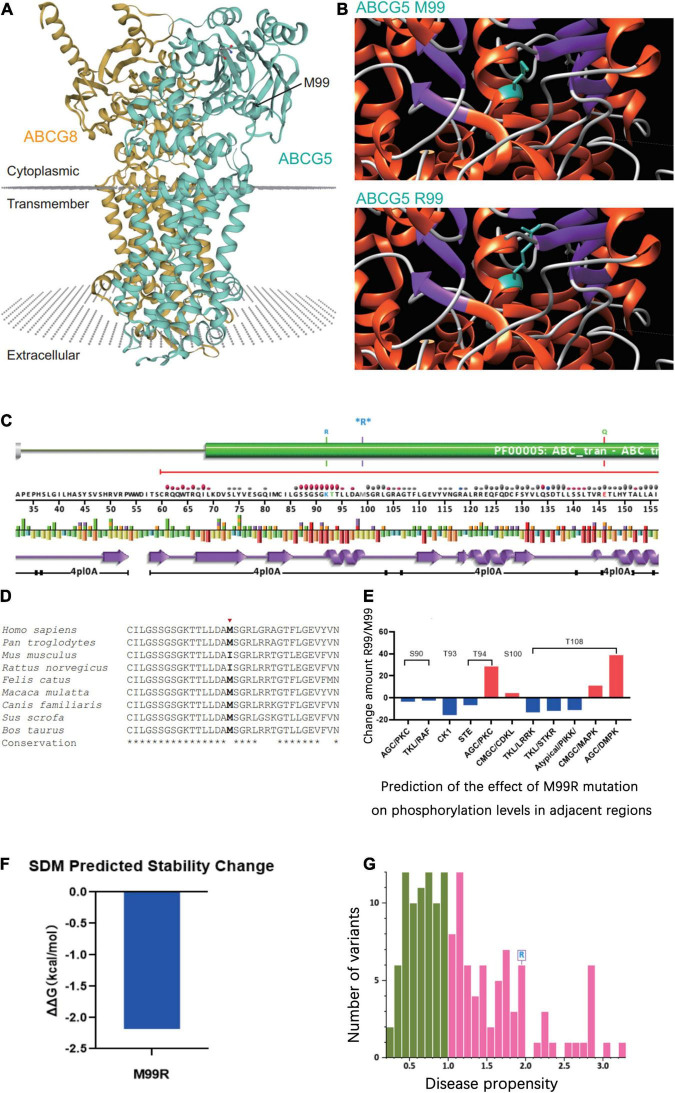
Prediction of the effect of the *ABCG5* (NM_022436) c.296 T >G (p.M99R) variant on the tertiary structure of *ABCG5*. **(A)** Tertiary structure of the *ABCG5*/*ABCG8* heterodimer (https://swissmodel.expasy.org/repository/uniprot/Q9H222?template=5do7). Green indicates *ABCG5* and orange indicates *ABCG8*. The position marked by the arrow indicates *ABCG5* p.M99R. **(B)** Prediction of changes in the tertiary structure of *ABCG5* after p.M99R mutation (green) by Chimera (https://community.chocolatey.org/packages/chimera/1.15). **(C)** Protein structural information at the p.M99R site. Functional prediction of the *ABCG5* M99R mutation (http://smart.embl-heidelberg.de/). **(D)** Conservation analysis of amino acid sequences near *ABCG5* M99R. **(E)** GPS 5.0 predicts the effect of M99R on phosphorylation modifications of nearby amino acid sequences (http://gps.biocuckoo.cn/). **(F)** DUET predicts the stability effect of M99R on *ABCG5* (http://structure.bioc.cam.ac.uk/duet). **(G)** Prediction of the pathogenicity of *ABCG5* M99R by VarSite (https://www.ebi.ac.uk/thornton-srv/databases/VarSite). The pink areas are all pathogenic mutations, and the higher the score of the number of variants, the stronger the pathogenicity.

**FIGURE 3:**
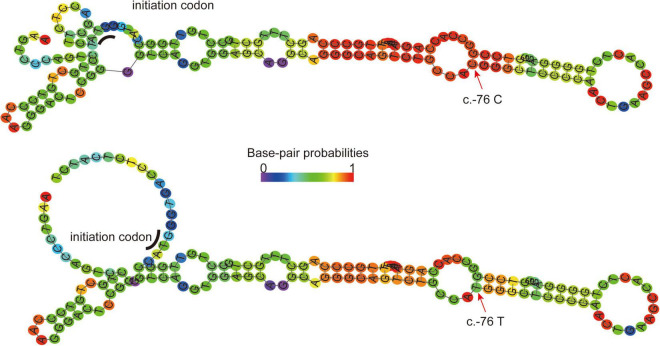
RNAFold (http://rna.tbi.univie.ac.at//cgi-bin/RNAWebSuite/RNAfold) predicts the secondary structure of ABCG mRNA fragments (−140 to + 17) in normal wild-type **(Top)** and C. − 76C >T mutations **(Bottom)**. Arrow, mutation site; Underline, translation initiation codon; Ruler color, possibility of matching. The c.−76C >T mutation significantly weakens the matching possibility of the stem-loop structure, leading to the apparent loosening of the RNA at the initiation codon, which frees the initiation codon and improves the translation efficiency. This is similar to the translation regulation mode of riboswitch.

The tertiary structure of the protein after the c.296 T >G p.M99R mutation of the *ABCG5* gene (NM_022436) was predicted by Swiss model software^1^ (Figure 2A)^2^ (Figure 2B),^3^ (Figure 2C). As a result, the spatial structure of the p.M99R position was found to change after the mutation. Aligning the same sequence of multiple mammalian species, it was observed that Met99 of the sterolin-1 protein encoded by the *ABCG5* gene was highly conserved in multiple species (Figure 2D). M99R affected the phosphorylation modification function of the CAMK pathway adjacent to T94, S100, and T108 (Figure 2E),^4^ which may have reduced the stability of the sterolin-1 protein structure exhibited a predicted increase in ΔΔG at M99R (2.21 Kcal/mol) (Figure 2F).^5^ The pink areas are all pathogenic mutations, and the higher the score of the number of variants, the stronger the pathogenicity. M99R was predicted to be pathogenic by VarSite (Figure 2G).^6^ The c.−76C >T mutation significantly weakens the matching possibility of the stem-loop structure, leading to the apparent loosening of the RNA at the initiation codon, which frees the initiation codon and improves the translation efficiency. This is similar to the translation regulation mode of riboswitch, suggesting that C. − 76 C >t affects the binding of transcriptional information or RNA binding proteins by regulating the structure of RNA, and ultimately affects gene transcription or RNA stability and translation (Figure 3).^7^

## Discussion

In Patel et al. mapped the sitosterolemia disease gene to two adjacent homologous genes on chromosome 2p21: *ABCG5* and *ABCG8*. Mutations in one of these two genes can lead to sitosterolemia, which is supported by *in vitro* data (7–9). To verify that the expression of *ABCG5* and *ABCG8* limits sterol absorption and promotes cholesterol excretion, Yu et al. established transgenic mice containing human *ABCG5* and *ABCG8* genes (10). Wild-type (*WT*) mice and *ABCG5* and *ABCG8* knockout mice (*G5G8*^–/–^ mice) were fed grain-based rodent chow containing 0.02% cholesterol and 4% fat *ad libitum*. The content of phytosterols in the plasma of wild-type mice was almost undetectable, whereas the absorption fraction of phytosterols in *G5G8*^–/–^ mice increased by 2-3 times, and phytosterols accumulated in the plasma and liver. The sitosterol content in the liver of *G5G8*^–/–^ mice increased significantly, and its plasma sitosterol level was approximately 30-fold higher than that of wild-type mice (11). In addition, Yu et al. fed *WT* mice and *G5G8*^–/–^ mice a powdered diet containing 0.02% cholesterol or 2% cholesterol, respectively, and determined the lipid content in plasma and liver by gas chromatography. The results showed that the plasma cholesterol level of *WT* mice did not increase significantly after a high cholesterol diet, while the liver cholesterol level increased by 3-fold. After a high cholesterol diet in *G5G8*^–/–^ mice, the plasma cholesterol increased by about 2.4-fold, while liver cholesterol levels increased by 18-fold (11). Thus, the overexpression of *ABCG5* and *ABCG8* resulted in a decrease in plasma phytosterol levels and an increase in bile cholesterol secretion. After blocking the pair of genes, the sterol levels in the plasma and liver increased significantly. Transgenic mice with increased ABCG5 and ABCG8 expression showed a 50% reduction in dietary sterol absorption, a significant increase in cholesterol bile secretion, and a significant reduction in plasma phytosterol levels. The overexpression or blockade of sterols in mice leads to changes in sterol content *in vivo*, confirming that *ABCG5* and *ABCG8* are related to the absorption and excretion of sterols.

Both *ABCG5* and *ABCG8* contain 13 exons and 12 introns, and are approximately 60 kb in size. Although the two genes are only 374 base pairs apart, they are transcribed in opposite directions and share a bidirectional promoter and regulatory elements (7). The proteins encoded by *ABCG5* and *ABCG8* are members of the ATP-binding cassette (ABC) transporter family. However, they are different from other transporters in the family because the transporters they encode are semi-transporters, sterolin-1 (encoded by *ABCG5*), and sterolin-2 protein (encoded by *ABCG8*). They contain six transmembrane structures and an ATP-binding site and form a heterodimer in the endoplasmic reticulum to become a complete ABC transporter. This is then transported to the apical membrane of hepatocytes and the apical membrane of intestinal brush border intestinal epithelial cells and is regulated by N-glycosylation and cAMP signaling during transport. It supplies energy through ATP at the apical membrane to regulate the absorption and excretion of phytosterols and cholesterol, especially sitosterol in phytosterols (12). Intestinal epithelial cells promote the excretion of phytosterols into the intestine, maintain the absorption rate of phytosterols at a low level of <5%, and promote the excretion of cholesterol to a certain extent. In liver cells, they promote the excretion of phytosterols and cholesterol into the bile, thereby reducing retention in the body (12–14).

Sitosterolemia, a genetic disease with autosomal mutations, has been confirmed to be caused by mutations in the *ABCG5* or *ABCG8* genes. The *ABCG8* mutation gene is mainly found in Caucasians, especially in Northern Europeans, while *ABCG5* gene mutations are mainly found in Asians, namely Chinese and Japanese (15). These two genes are located in close proximity and are homologous; however, the mutation of *ABCG8* is more polymorphic than that of *ABCG5* (16). As of April 2022, there were 44 (115) and 48 (119) related mutations in *ABCG5* or *ABCG8* genes included in HGMD, mainly missense mutations, while some were splicing mutations and base deletion frameshift mutations. Monkol Lek et al. analyzed the exon sequences of 60,706 people and found that more than 110 cases of *ABCG5* and *ABCG8* carried loss-of-function alleles, of which only 37 had been described as sitosterolemia. At the same time, nearly 1990 missense variants were found; however, the pathogenicity of these missense mutations was uncertain and had not been tested or clinically verified (6). The potentially pathogenic c.296T >G (p.M99R) variant, identified in the sitosterolemia family, was a novel variant. It has been reported that *ABCG5* c.293c >G (p.Ala98Gly) (CM169032) has a disease spectrum of congenital giant thrombocytopenia with moderate thrombocytopenia (17). It can be seen that *ABCG5* or *ABCG8* gene mutations can lead to a group of highly heterogeneous disease spectrum with different phenotypes. As far as the currently confirmed mutations disease spectrum is concerned, sitosterolemia, phytosterolemia, macrothrombocytopaenia, cholelithiasis, coronary artery disease, increased risk, hypercholesterolemia, increased serum cholesterol, in low-cholesterol consumers, reduced serum non-cholesterol sterols, and smaller decline in cholesterol synthesis after weight loss, among others, are the most common, followed by phytosterolemia.

Mutations in *ABCG5* or *ABCG8* cause proteins encoding the transporters sterolin-1 and sterolin-2 to fail to form stable heterodimers, resulting in hypersteroidemia. The levels of phytosterols in the plasma, lipoprotein, red blood cells, adipose tissue, and skin surface are increased in patients with sitosterolemia. The main clinical manifestations are tendon and skin xanthoma (18). This can occur when the patient is very young, such as during the first year after birth (19). The main mechanism of xanthoma appears to be similar to the early developmental stage of atherosclerosis, that is, the formation of foam cell populations, especially in areas prone to friction (14, 20). According to statistical analysis of familial hypercholesterolemia patients, patients with xanthoma are 3.2-fold more likely to develop cardiovascular disease than patients without xanthoma (21). Furthermore, the accumulation of phytosterols in plasma lipoproteins can increase tissue sterols by affecting the stability of lipoprotein cholesterol and phytosterols. This triggers an inflammatory response, promotes the formation of foam cells and plaques, and induces premature atherosclerosis and coronary heart disease (22). In addition, patients with sitosterolemia may also have anemia and hemolysis. The lifespan of erythrocytes is closely related to lipid metabolism in the plasma, which is related to the abnormal morphology of erythrocytes and the increase in osmotic fragility caused by the appearance of phytosterols in erythrocytes (23). Patients with sitosterolemia also experience abnormal bleeding and thrombocytopenia. This is related to the direct entry of phytosterols into the platelet membrane, resulting in decreased αIIbβ3 expression on the membrane surface, which in turn leads to the loss of GPIba-FlnA linkages, the formation of microparticles, and platelet dysfunction (24), making them more susceptible to deformity and rupture. In addition, this can also be related to joint pain, arthritis, splenomegaly, adrenal dysfunction, and other clinical manifestations.

Hayato Tada suggested that sitosterolemia can be diagnosed if the following conditions are all met at the same time (25): (1) clinical manifestations (xanthoma of skin or tendon); (2) laboratory tests (serum sitosterol ≥1 mg/dl); (3) familial hypercholesterolemia and cerebrotendinous xanthomatosis are excluded; (4) pathogenic mutations *ABCG5* or *ABCG8*. But the clinical manifestations of sitosterolemia are highly heterogeneous, although the genotype and phenotype lack an obvious correlation, and most laboratories lack conditions for phytosterol determination (26). Thus, at present, the diagnosis of this disease remains very difficult. Some patients with sitosterolemia have no xanthoma and only show blood system changes, such as hemolytic anemia and thrombocytopenia. This is often misdiagnosed as idiopathic thrombocytopenia and EVANS syndrome, or even splenectomy (27). Therefore, for the diagnosis of sitosterolemia, it is not only necessary to proceed from clinical manifestations but also to improve the detection of plasma phytosterols, the morphological analysis of blood cells, and genetic diagnosis (27). And based on the pathophysiology of sitosterolemia,ezetimibe and low phytosterols diet are currently effective methods for the treatment of sitosterolemia. Ezetimibe, a Niemann-Pick C1 Like 1 inhibitor, can effectively reduce plasma phytosterols and cholesterol in patients with sitosterolemia by affecting the absorption of various sterols in the intestine (25, 28–30).

## Data Availability Statement

The original contributions presented in the study are included in the article/supplementary materials, further inquiries can be directed to the corresponding authors.

## Ethics Statement

The studies involving human participants were reviewed and approved by the Ethics Committee of Fujian Provincial Hospital. Written informed consent to participate in this study was provided by the participants’ legal guardian/next of kin. Written informed consent was obtained from the individual(s), and minor(s)’ legal guardian/next of kin, for the publication of any potentially identifiable images or data included in this article.

## Author Contributions

J-WL and X-FL designed the study. M-FS, Y-NH, W-XC, and MW performed the data collection. Q-YW, J-HZ, and Y-PZ performed the data analysis and interpretation of results. M-FS wrote the original draft. J-WL and L-SL reviewed and provided critical comments on the manuscript. All authors read and approved the final version of the manuscript.

## Conflict of Interest

The authors declare that the research was conducted in the absence of any commercial or financial relationships that could be construed as a potential conflict of interest.

## Publisher’s Note

All claims expressed in this article are solely those of the authors and do not necessarily represent those of their affiliated organizations, or those of the publisher, the editors and the reviewers. Any product that may be evaluated in this article, or claim that may be made by its manufacturer, is not guaranteed or endorsed by the publisher.

## Footnotes

1
https://swissmodel.expasy.org/repository/uniprot/Q9H222?template=5do7

2
https://community.chocolatey.org/packages/chimera/1.15

3
http://smart.embl-heidelberg.de/

4
http://gps.biocuckoo.cn/

5
http://structure.bioc.cam.ac.uk/duet

6
https://www.ebi.ac.uk/thornton-srv/databases/VarSite

7
https://genome.ucsc.edu/cgi-bin/hgTracks?db=hg19
